# Draft Genome Sequences of Three Multidrug-Resistant Staphylococcus spp. Isolated from Hospital Wastewater in Malaysia

**DOI:** 10.1128/MRA.00332-21

**Published:** 2021-05-06

**Authors:** Nurul Syazwani Ahmad Sabri, Siti Norayuni Mohd Zulkeflle, Nurtasbiyah Yusof, Fazrena Nadia Md Akhir, Nor’azizi Othman, Zuriati Zakaria, Hirofumi Hara

**Affiliations:** a Department of Chemical and Environmental Engineering, Malaysia-Japan International Institute of Technology, Universiti Teknologi Malaysia, Kuala Lumpur, Malaysia; b Department of Mechanical Precision Engineering, Malaysia-Japan International Institute of Technology, Universiti Teknologi Malaysia, Kuala Lumpur, Malaysia; University of Rochester School of Medicine and Dentistry

## Abstract

Staphylococcus spp. are Gram-positive bacteria that reside within the normal microbiota of humans and animals but pose a health threat as reservoirs of antimicrobial resistance genes. Here, we present the draft genome sequences of three Staphylococcus sp. strains isolated from hospital wastewater in Malaysia that demonstrated resistance to multiple antibiotics.

## ANNOUNCEMENT

Staphylococcus spp. have emerged as a major health threat in nosocomial and community settings due to their active participation in the transmission of antibiotic resistance (1–3). Water contaminated with antibiotics from human waste, livestock farms, health care settings, and pharmaceutical residues may serve as a risk factor that places Southeast Asia at the highest risk for the emergence and spread of antibiotic resistance in humans (4–6). Here, we isolated and sequenced three Staphylococcus sp. strains from hospital wastewater to discover the mechanisms and mobile genetic elements that are responsible for the transmission of resistance genes in the tropics. Wastewater samples were collected from a river near the sewage treatment plant of the University of Malaya Medical Centre (UMMC) (3°07′01.5″N, 101°39′51.4″E) in Kuala Lumpur, Malaysia. The samples were filtered and grown on mannitol salt agar (MSA) at 37°C for 24 h, followed by Gram staining and catalase and coagulase tests (7). The genus and species were confirmed after 16S rRNA gene sequencing using the 27F and 1492R primers, as well as the *nuc* primer (which encodes a thermonuclease enzyme in certain Staphylococcus aureus isolates). Isolates were then screened for multidrug-resistant (MDR) strains using the disk diffusion assay (8, 9). Staphylococcus sp. strains S36, S59, and S75 showed resistance to multiple classes of antibiotics and were selected for whole-genome sequencing. Genomic DNA of the strains was extracted from pure cultures grown overnight in nutrient broth using the HiYield genomic DNA minikit (RBC BioScience). A 400-bp library was constructed using the Ion Xpress Plus fragment library kit (Thermo Fisher Scientific) according to the manufacturer’s protocol and quantified using an Agilent 2100 bioanalyzer. The library was diluted prior to template preparation using the Ion Chef system (Thermo Fisher Scientific), followed by sequencing using the Ion S5XL system (Thermo Fisher Scientific). Torrent Suite software (Thermo Fisher Scientific) was used for raw data analysis, alignment, and variant calling. Short reads from the Ion S5XL system were quality trimmed and assembled using CLC Genomics Workbench v11.0.1. The reads were trimmed with the following parameters: quality score limit, 0.05; discarded reads, <400 nucleotides; and maximum number of ambiguous nucleotides, 2. Default parameters were used for the assembly. Assembly metrics were evaluated using QUAST v5.0.2 (10). Genomic features were annotated with the NCBI PGAP v5.1 (11) and RAST v2.0 (12) (Table 1). Antimicrobial resistance gene sequences were identified with AMRFinder v3.9.8 (13).

**TABLE 1:** Genomic features of Staphylococcus sp. strains S36, S59, and S75

Strain	No. of reads	Genome size (bp)	No. of contigs	*N*_50_ (bp)	G+C content (%)	No. of CDS^*a*^	No. of RNAs	No. of tRNAs	SRA accession no.
S36	2,051,709	2,722,586	123	61,752	32.4	2,510	7	60	SRR13398928
S59	1,995,217	2,690,341	104	123,494	32.4	2,507	6	60	SRR13398927
S75	3,190,974	2,713,405	108	74,768	32.4	2,501	11	63	SRR13398926

a CDS, coding DNA sequences.

Based on the RAST annotation for each strain, numerous enzymes, including TetR family regulatory proteins of the MDR cluster, β-lactamase, and DNA gyrase subunits A and B, were predicted to be involved in the resistance mechanisms. AMRFinder identified genes that confer resistance to β-lactam (*bla*), fusidic acid (*fusF*), macrolide [*erm*(C) and *abc-f*], rifamycin (*arr*), and quaternary ammonium (*qacC* and *qacCGHJ*). The genome information of Staphylococcus sp. strains from tropical climates compared with isolates from temperate countries will greatly contribute to essential surveillance data for antibiotic resistance, which will be useful for developing new compounds and modifying older agents that retain potent activity against target pathogens.

### Data availability.

The draft genome sequences of all three Staphylococcus sp. strains were deposited in GenBank under BioProject number PRJNA689868. The NCBI assembly numbers for strains S36, S59, and S75 are GCA_016722985.1, GCA_016723005.1, and GCA_016722825.1, respectively. The SRA numbers are provided in Table 1.
